# FORMAL AND INFORMAL VOLUNTEERING AMONG OLDER ADULTS: ASSOCIATIONS WITH HEALTH OUTCOMES ACROSS SEXUAL ORIENTATIONS

**DOI:** 10.1093/geroni/igac059.1836

**Published:** 2022-12-20

**Authors:** Joseph Frey, Huei-Wern Shen, Yi Wang

**Affiliations:** University of North Texas, Denton, Texas, United States; University of North Texas, Denton, Texas, United States; University of Iowa, Iowa City, Iowa, United States

## Abstract

Volunteering is associated with positive physical and mental health outcomes among older adults. However, little is known about the impact of volunteering on health among older lesbian, gay, and bisexual (LGB) Americans. Using nationally representative 2016 Health and Retirement Study data, the present study explored associations between volunteering and health outcomes (i.e., self-rated health, current memory rating) across sexual orientations among older Americans. Two types of volunteering were considered in the study—formal volunteering (doing unpaid work for religious, educational, health-related, or other charitable organizations) and informal volunteering (providing unpaid help to friends, neighbors, or relatives who do not co-reside). We included 235 (5.7%) respondents who self-identified as LGB or something else, and 3,894 (94.3%) heterosexual-identified respondents. Controlling for health-related variables (e.g., ADLs/IADLs), SES (e.g., income), contextual features (e.g., urban/rural), and sociodemographics (e.g., age, gender, race), results from eight stratified OLS regression models showed that for heterosexual-identified older adults, both formal and informal volunteering promoted self-rated health (p<.001, respectively) and memory rating (p<.001, respectively). However, for LGB-identified older adults, only informal volunteering was detected to increase memory rating (p<.038). Findings suggest that the well-documented health benefits of formal volunteering may not be supported in the LGB population. The current theoretical model and discourse about productive aging should be modified to be more inclusive with respect to sexual orientation. Further research identifying the pathways (e.g., identity-related stigmatization and discrimination) between various types of volunteering and health outcomes among older LGB adults is urged and possible directions will be discussed.

